# Inequalities for the fractional convolution operator on differential forms

**DOI:** 10.1186/s13660-018-1768-x

**Published:** 2018-07-16

**Authors:** Zhimin Dai, Huacan Li, Qunfang Li

**Affiliations:** 1 School of Science, Xi’an Technological University, Xi’an, China; 2 School of Science, Jiangxi University of Science and Technology, Ganzhou, China; 3 Department of Mathematics, Ganzhou Teachers’ College, Ganzhou, China

**Keywords:** Fractional convolution operator, Coifman type inequality, BMO norm, Lipschitz norm, Differential form

## Abstract

The purpose of this paper is to derive some Coifman type inequalities for the fractional convolution operator applied to differential forms. The Lipschitz norm and BMO norm estimates for this integral type operator acting on differential forms are also obtained.

## Introduction

As a generalization of functions, the differential form can be regarded as a special kind of vector-valued function. So, if some operators in function spaces are generalized to that in differential forms, similar properties could be obtained as in the function space. In recent years, the research on the generalization of operators from functional spaces to differential forms seems to become a new highlight in the inequalities with differential forms, see [1–6]. In this paper, we mainly consider the following convolution type fractional integrals operator acting on differential forms and develop some norm inequalities for the fractional convolution operator. Given a nonnegative, locally integrable function $K_{\alpha }$ and $\hbar _{I}(y)$ is a bounded function with a compactly supported set on $\mathbb{R}^{n}$, write $\hbar _{I}(y)\in L^{\infty }_{c}$. The fractional convolution operator $F_{\alpha }$ is defined by a convolution integral

1.1 $$ F_{\alpha }\hbar (x)=\sum_{I} \biggl( \int _{\mathbb{R}^{n}}K_{\alpha }(x-y)\hbar _{I}(y)\,\mathrm{d}y \biggr)\,\mathrm{d}x_{I}, $$

provided this integral exists for almost all $\mathbb{R}^{n}$, where $\hbar (x)=\sum_{I}\hbar _{I}(y)\,\mathrm{d}x_{I}$ is a *ℓ*-form defined on $\mathbb{R}^{n}$, the summation is over all ordered *ℓ*-tuples *I*. The function $K_{\alpha }$ is also assumed to be a wide class of kernels satisfying the following growth condition (see [7]):

1. $K_{\alpha }\in S_{\alpha }$ if there exists a constant $C>0$ such that
  1.2 $$ \int _{\vert x \vert \sim s} \bigl\vert K_{\alpha }(x) \bigr\vert \, \mathrm{d}x\leq Cs^{\alpha }; $$
2. $K_{\alpha }$ is said to satisfy the $L^{\alpha,\varphi }$-Hörmander condition, and write $K_{\alpha }\in H_{\alpha,\varphi }$. If there exist $c\geq 1$ and $C>0$ (only dependent on *φ*) such that, for all $y\in \mathbb{R}^{n}$ and $R>c\vert y \vert $,
  1.3 $$ \sum^{\infty }_{m=1} \bigl(2^{m}R \bigr)^{n-\alpha }\nparallel K_{\alpha }(\cdot -y)-K_{\alpha }(\cdot )\bigr\Vert _{\varphi (\vert x \vert \sim 2^{m}R)}\leq C, $$
  where *φ* is a Young function defined on $[0,+\infty )$, $\vert x \vert \sim s$ stands for the set $\{s<\vert x \vert \leq 2s\}$, $O(0,s)$ is a ball with the center at the origin and the radius equal to *s*, and the *φ*-mean Luxemburg norm of a function *f* on a cube (or a ball)*O* in $\mathbb{R}^{n}$ is given by
  1.4 $$ \nparallel f\Vert _{\varphi (O)} =\inf \biggl\{ \lambda >0:\frac{1}{\vert O \vert } \int _{O}\varphi \biggl(\frac{\vert f \vert }{\lambda } \biggr)\,\mathrm{d}x \leq 1 \biggr\} . $$

Differential forms can be viewed as an extension of functions. When $\hbar (x)$ is a 0-form, the above-mentioned notations are in accord with those of function spaces, and the fractional convolution operator $F_{\alpha }$ we study in this paper degenerates into the operator which Bernardis discussed in [7]. Namely, for any Lebesgue measurable function $f\in L^{\infty }_{c}$, $F_{\alpha }$ is given as follows:

1.5 $$ F_{\alpha }f(x)= \int _{\mathbb{R}^{n}}K_{\alpha }(x-y)f(y)\,\mathrm{d}y. $$

This degenerated fractional convolution operator was also introduced by Riveros in [8], who presented weighted Coifman type estimates, two weight estimates of strong and weak type for general fractional operators and gave applications to fractional operators produced by a homogeneous function and a Fourier multiplier.

Now we introduce some notations and definitions. Let Θ be an open subset of $\mathbb{R}^{n}$ ($n\geq 2$) and *O* be a ball in $\mathbb{R}^{n}$. Let *ρO* denote the ball with the same center as *O* and $\operatorname{diam}(\rho O) = \rho \operatorname{diam}(O)(\rho >0)$. $\vert \Theta \vert $ is used to denote the Lebesgue measure of a set $\Theta \subset \mathbb{R}^{n}$. Let $\bigwedge^{\ell }= \bigwedge^{\ell }(\mathbb{R}^{n}), \ell = 0,1,\ldots, n$, be the linear space of all *ℓ*-forms $\hbar (x)= \sum_{I}\hbar _{I}(x)\,\mathrm{d}x_{I}=\sum_{I}\hbar _{i_{1}i_{2}\cdots i_{\ell }}(x)\,\mathrm{d}x_{i_{1}}\wedge\mathrm{d}x_{i_{2}}\cdots \wedge\mathrm{d}x_{i_{\ell }}$ in $\mathbb{R}^{n}$, where $I= (i_{1}, i_{2}, \ldots,i_{\ell }), 1\leq i_{1}< i_{2}<\cdots <i_{\ell } \leq n$, are the ordered *ℓ*-tuples. Moreover, if each of the coefficients $\hbar _{I}(x)$ of $\hbar (x)$ is differential on Θ, then we call $\hbar (x)$ a differential *ℓ*-form on Θ and use $D^{\prime}(\Theta,\bigwedge^{\ell })$ to denote the space of all differential *ℓ*-forms on Θ. $C^{\infty }(\Theta,\bigwedge^{\ell })$ denotes the space of smooth *ℓ*-forms on Θ. The exterior derivative $d:D^{\prime}(\Theta,\bigwedge^{\ell })\rightarrow D^{\prime}(\Theta,\bigwedge^{\ell +1})$, $\ell =0,1,\ldots,n-1$, is given by

1.6 $$ d\hbar (x)=\sum_{I}\sum ^{n}_{j=1}\frac{\partial \hbar _{i_{1}i_{2}\cdots i_{\ell }}(x)}{\partial x_{j}}\,\mathrm{d}x_{j} \wedge\mathrm{d}x_{i_{1}}\wedge\mathrm{d}x_{i_{2}}\wedge \cdots \wedge\mathrm{d}x_{i_{\ell }} $$

for all $\hbar \in D^{\prime}(\Theta,\bigwedge^{\ell })$. $L^{p}(\Theta,\bigwedge^{\ell })(1\leq p<\infty )$ is a Banach space with the norm $\Vert \hbar \Vert _{p,\Theta }=(\int _{\Theta }\vert \hbar (x) \vert ^{p}\,\mathrm{d}x)^{1/p}=(\int _{\Theta }(\sum_{I}\vert \hbar _{I}(x) \vert ^{2})^{p/2}\,\mathrm{d}x)^{1/p}<\infty $. Similarly, the notations $L^{p}_{\textrm{loc}}(\Theta,\bigwedge^{\ell })$ and $W^{1,p}_{\textrm{loc}}(\Theta,\bigwedge^{\ell })$ are self-explanatory.

From [9], *ħ* is a differential form in a bounded convex domain Θ, then there is a decomposition

1.7 $$ \hbar =d(T\hbar )+T(d\hbar ), $$

where *T* is called a homotopy operator. For the homotopy operator *T*, we know that

1.8 $$ \Vert T\hbar \Vert _{p,O}\leq C\vert O \vert \operatorname{diam}(O)\Vert \hbar \Vert _{p,O} $$

holds for any differential form $\hbar \in L^{p}_{\textrm{loc}}(\Theta,\bigwedge^{\ell }),\ell =1,2,\ldots,n,1< p<\infty $. Furthermore, we can define the *ℓ*-form $\hbar _{\Theta } \in D^{\prime} (\Theta,\bigwedge^{\ell }) $ by

1.9 $$ \hbar _{\Theta }= \textstyle\begin{cases} \vert \Theta \vert ^{-1}\int _{\Theta }\hbar (x)\,\mathrm{d}x, &\ell =0, \\ dT(\hbar ), & \ell =1,2,\ldots,n, \end{cases} $$

for all $\hbar \in L^{p}(\Theta,\bigwedge^{\ell }),1\leq p<\infty $.

A non-negative function $w\in L^{1}_{\textrm{loc}}(\mathrm{d}x)$ is called a weight. We recall the definitions of the Muckenhoupt weights and the reverse Hölder condition (see [10]). For $1< p<\infty $, we say that $w\in \mathcal{A}_{p}$ if there exists a constant $C>0$ such that, for every ball $O\subset \mathbb{R}^{n}$,

1.10 $$ \biggl(\frac{1}{\vert O \vert } \int _{O}w\,\mathrm{d}x \biggr) \biggl(\frac{1}{\vert O \vert } \int _{O}w^{-\frac{1}{p-1}}\,\mathrm{d}x \biggr)^{p-1} \leq C . $$

For the case $p=1$, $w\in \mathcal{A}_{1}$ if there exists a constant $C>0$ such that, for every ball $O\subset \mathbb{R}^{n}$,

1.11 $$ \frac{1}{\vert O \vert } \int _{O}w\,\mathrm{d}x\leq C \operatorname{ess}\inf_{x\in O}w(x). $$

Also $\mathcal{A}_{\infty }=\bigcup _{p\geq 1}\mathcal{A}_{p}$. It is well known that $\mathcal{A}_{p}\subset \mathcal{A}_{q}$ for all $1\leq p\leq q\leq \infty $, and also that for $1< p\leq \infty $, if $w \in \mathcal{A}_{p}$, then there exists $\varepsilon >0$ such that $w\in \mathcal{A}_{p-\varepsilon }$.

A function $\varphi : [0,\infty )\rightarrow [0,\infty )$ is a Young function if it is continuous, convex, increasing and satisfies $\varphi (0)=0$ and $\varphi (t)\rightarrow \infty $ as $t\rightarrow \infty $. Each Young function *φ* has an associated complementary Young function *φ̄* satisfying

1.12 $$ t\leq \varphi ^{-1}(t)\bar{\varphi }^{-1}(t)\leq 2t $$

for all $t>0$, where $\varphi ^{-1}(t)$ is the inverse function of $\varphi (t)$ (see [11]).

For each locally integrable function *f* and $0 \leq \alpha < n$, the fractional maximal operator associated with the Young function *φ* is defined by

1.13 $$ M_{\alpha,\varphi }f(x)=\sup_{x\in O}\vert O \vert ^{\frac{\alpha }{n}}\nparallel f\Vert _{\varphi (O)}. $$

For $\alpha =0$, we write $M_{\varphi }$ instead of $M_{0,\varphi }$. When $\varphi (t)=t$, then $M_{\alpha,\varphi }=M_{\alpha }$ is the classical fractional maximal operator. For $\alpha =0$ and $\varphi (t)=t$, we obtain $M_{0,\varphi }=M$ is the Hardy–Littlewood maximal operator (see [8]).

## The Coifman type inequalities for the fractional convolution operator

In [7], the inequality for the fractional convolution operator in function with the fractional maximal operator, that is, the Coifman type inequality, is proved.

### Lemma 2.1

*Let* *φ* *be a Young function on* $[0,+\infty )$ *and* *f* *be any* *n*-*tuple function on* $\mathbb{R}^{n}$ *with* $f\in L^{\infty }_{c}$. *Suppose that the fractional convolution operator* $F_{\alpha }=K_{\alpha }\ast f$ *and its kernel satisfies* $K_{\alpha }\in S_{\alpha }\cap H_{\alpha,\varphi }$, *where* $0<\alpha <n$. *Then there exists a constant* *C* *such that*

2.1 $$ \int _{\mathbb{R}^{n}} \bigl\vert F_{\alpha }f(x) \bigr\vert ^{p}w(x)\,\mathrm{d}x\leq C \int _{\mathbb{R}^{n}} \bigl[M_{\alpha,\bar{\varphi }}f(x) \bigr]^{p}w(x)\,\mathrm{d}x $$

*for any* $0< p<\infty $ *and* $w\in A_{\infty }$.

### Theorem 2.1

*Let* *φ* *be a Young function on* $[0,+\infty )$ *and* *f*, *g* *be two functions defined on* $\mathbb{R}^{n}$ *with* $\vert f(x) \vert \leq \vert g(x) \vert $ *for all* $x\in \mathbb{R}^{n} $. *Then for all cubes* *O* *and the Young functions* *φ*,

2.2 $$ \nparallel f\Vert _{\varphi (O)} \leq \nparallel g \Vert _{\varphi (O)}. $$

### Proof

Since *φ* is a Young function, it follows that

2.3 $$\begin{aligned} &\frac{1}{\vert O \vert } \int _{O}\varphi \biggl(\frac{\vert f \vert }{\nparallel g\Vert _{\varphi (O)}} \biggr)\,\mathrm{d}x \leq \frac{1}{\vert O \vert } \int _{O}\varphi \biggl(\frac{\vert g \vert }{\nparallel g \Vert _{\varphi (O)}} \biggr)\,\mathrm{d}x \leq 1 \\ &\quad \Rightarrow \quad \nparallel g\Vert _{\varphi (O)}\in E= \biggl\{ \lambda >0: \frac{1}{\vert O \vert } \int _{O}\varphi \biggl(\frac{\vert f \vert }{\lambda } \biggr)\,\mathrm{d}x \leq 1 \biggr\} \\ & \quad \Rightarrow \quad \nparallel f\Vert _{\varphi (O)}=\inf E\leq \nparallel g\Vert _{\varphi (O)}. \end{aligned}$$

 □

According to Theorem 2.1, we can get a similar conclusion to Lemma 2.1.

### Theorem 2.2

*Let* *φ* *be a Young function on* $[0,+\infty )$, $\hbar =\sum_{I}\hbar _{I}\,\mathrm{d}x_{I}$ *be a differential form on* $\Theta \subset \mathbb{R}^{n}$, *and let all the ordered* *ℓ*-*tuples* *I* *satisfy* $\hbar _{I}\in L^{\infty }_{c}$. *Suppose that* $F_{\alpha }$ *is a fractional convolution operator applied to differential forms and its kernel function* $K_{\alpha }$ *satisfies* $K_{\alpha }\in S_{\alpha }\cap H_{\alpha,\varphi }$, *where* $0<\alpha <n$. *Then there exists a constant* *C* *such that*

2.4 $$ \int _{\mathbb{R}^{n}} \bigl\vert F_{\alpha }\hbar (x) \bigr\vert ^{p}w(x)\,\mathrm{d}x\leq C \int _{\mathbb{R}^{n}} \bigl[M_{\alpha,\bar{\varphi }}\hbar (x) \bigr]^{p}w(x)\,\mathrm{d}x $$

*for any* $0< p<\infty $ *and* $w\in A_{\infty }$.

### Proof

By Lemma 2.1 and the following basic inequality

2.5 $$ \sum^{n}_{i=1}\vert a_{i} \vert ^{s}\leq n \Biggl(\sum^{n}_{i=1} \vert a_{i} \vert \Biggr)^{s}\leq n^{s+1}\sum ^{n}_{i=1}\vert a_{i} \vert ^{s}, $$

where $s>0$ is any constant, it follows that

2.6 $$\begin{aligned} \Vert F_{\alpha }\hbar \Vert ^{p}_{p,w,\mathbb{R}^{n}} & =\int _{\mathbb{R}^{n}} \bigl\vert F_{\alpha }\hbar (x) \bigr\vert ^{p}w(x)\,\mathrm{d}x \\ & =\int _{\mathbb{R}^{n}} \biggl( \sum_{I}\biggl( \int _{\mathbb{R}^{n}}K_{\alpha }(x-y)\hbar _{I}(y)\,\mathrm{d}y \biggr) ^{2} \biggr) ^{p/2}w(x)\,\mathrm{d}x \\ & \leq\int _{\mathbb{R}^{n}}C_{1}\sum_{I} \biggl( \int _{\mathbb{R}^{n}}K_{\alpha }(x-y)\hbar _{I}(y)\,\mathrm{d}y \biggr) ^{p}w(x)\,\mathrm{d}x \\ & =C_{1}\sum_{I}\int _{\mathbb{R}^{n}} \biggl(\int _{\mathbb{R}^{n}}K_{\alpha }(x-y)\hbar _{I}(y)\,\mathrm{d}y \biggr) ^{p}w(x)\,\mathrm{d}x \\ & \leq C_{2}\sum_{I}\int _{\mathbb{R}^{n}} \bigl[M_{\alpha,\bar{\varphi }}\hbar _{I}(x) \bigr]^{p}w(x)\,\mathrm{d}x \\ & =C_{2} \int _{\mathbb{R}^{n}}\sum_{I} \bigl[M_{\alpha,\bar{\varphi }}\hbar _{I}(x) \bigr]^{p}w(x)\,\mathrm{d}x \\ & \leq C_{3}\int _{\mathbb{R}^{n}} \biggl( \sum_{I}M_{\alpha,\bar{\varphi }} \hbar _{I}(x) \biggr) ^{p}w(x)\,\mathrm{d}x. \end{aligned}$$

Then, by the definition of the fractional maximal operator, notice that for any *I* such that $\vert \hbar _{I} \vert \leq \vert \hbar \vert $, we obtain that

2.7 $$\begin{aligned} &\sum_{I}M_{\alpha,\bar{\varphi }}\hbar _{I}(x) \\ & \quad =\sum_{I}\sup_{x\in O} \vert O \vert ^{\alpha /n}\nparallel \hbar _{I}\bigr\Vert _{\bar{\varphi }(O)} \\ & \quad \leq C^{\ell }_{n}\sup_{x\in O}\vert O \vert ^{\alpha /n}\sum_{I}\nparallel \hbar_{I}\bigr\Vert _{\bar{\varphi }(O)} \\ & \quad \leq C^{\ell }_{n}\sup_{x\in O}\vert O \vert ^{\alpha /n}C^{\ell }_{n}\nparallel \hbar \Vert _{\bar{\varphi }(O)} \\ & \quad \leq C_{4}M_{\alpha,\bar{\varphi }}\hbar (x). \end{aligned}$$

Combining (2.6) and (2.7), we have

2.8 $$\begin{aligned} &\Vert F_{\alpha }\hbar \Vert ^{p}_{p,w,\mathbb{R}^{n}} \\ & \quad \leq C_{3} \int _{\mathbb{R}^{n}} \biggl( \sum_{I}M_{\alpha,\bar{\varphi }} \hbar _{I}(x) \biggr) ^{p}w(x)\,\mathrm{d}x \\ & \quad \leq C_{3} \int _{\mathbb{R}^{n}} \bigl(C_{4}M_{\alpha,\bar{\varphi }}\hbar (x) \bigr)^{p}w(x)\,\mathrm{d}x \\ & \quad \leq C_{5} \int _{\mathbb{R}^{n}} \bigl(M_{\alpha,\bar{\varphi }}\hbar (x) \bigr)^{p}w(x)\,\mathrm{d}x. \end{aligned}$$

 □

### Theorem 2.3

*Let* *φ* *be a Young function on* $[0,+\infty )$, $\hbar =\sum_{I}\hbar _{I}\,\mathrm{d}x_{I}$ *be a differential form on* $\Theta \subset \mathbb{R}^{n}$, *and for all the ordered* *ℓ*-*tuples*,*let* *I* *satisfy* $\hbar _{I}\in L^{\infty }_{c}$. *Suppose that* $F_{\alpha }$ *is a fractional convolution operator on differential forms and its kernel function* $K_{\alpha }$ *satisfies* $K_{\alpha }\in S_{\alpha }\cap H_{\alpha,\varphi }$, *where* $0<\alpha <n$. *Then there exists a constant* *C* *such that*

2.9 $$ \int _{O} \bigl\vert F_{\alpha }\hbar (x) \bigr\vert ^{p}\,\mathrm{d}x\leq C \int _{O} \bigl[M_{\alpha,\bar{\varphi }}\hbar (x) \bigr]^{p}\,\mathrm{d}x $$

*for any* $0< p<\infty $ *and all the balls* $O\subset \mathbb{R}^{n}$.

### Proof

By the definition of the $\mathcal{A}_{\infty }$-weight, there exist $r_{0}\geq 1$ and a constant $C<\infty $ such that, for all the balls $O\subset \mathbb{R}^{n}$, it follows that

2.10 $$ \biggl( \frac{1}{{\vert O \vert }} \int _{O}w(x)\,\mathrm{d}x \biggr) \biggl( \frac{1}{{\vert O \vert }} \int _{O}w(x)^{-\frac{1}{r_{0}-1}}\,\mathrm{d}x \biggr) ^{r_{0}-1}\leq C. $$

With the arbitrariness of the condition $w\in \mathcal{A}_{\infty }$ of Theorem 2.2, now get any ball $O_{0}\subset \mathbb{R}^{n}$ and let

$$ w(x)=\chi _{O_{0}}(x)= \textstyle\begin{cases} 1, & x \in O_{0}; \\ 0, & x \notin O_{0}. \end{cases} $$

It is easy to check that $w(x)=\chi _{O_{0}}(x)$ satisfies (2.10). In fact, we have

2.11 $$\begin{aligned} & \biggl( \frac{1}{{\vert O \vert }} \int _{O}\chi _{O_{0}}(x)\,\mathrm{d}x \biggr) \biggl( \frac{1}{{\vert O \vert }} \int _{O}\chi _{O_{0}}(x)^{-\frac{1}{r_{0}-1}}\,\mathrm{d}x \biggr) ^{r_{0}-1} \\ & \quad = \biggl( \frac{1}{{\vert O \vert }}\vert O\cap O_{0} \vert \biggr) \biggl( \frac{1}{{\vert O \vert }}\vert O\cap O_{0} \vert \biggr) ^{r_{0}-1} \\ & \quad = \biggl( \frac{1}{{\vert O \vert }}\vert O\cap O_{0} \vert \biggr) ^{r_{0}}\leq 1. \end{aligned}$$

Thus

2.12 $$\begin{aligned} & \int _{O_{0}} \bigl\vert F_{\alpha }\hbar (x) \bigr\vert ^{p}\,\mathrm{d}x \\ & \quad = \int _{\mathbb{R}^{n}} \bigl\vert F_{\alpha }\hbar (x) \bigr\vert ^{p}\chi _{O_{0}}(x)\,\mathrm{d}x \\ & \quad \leq C \int _{\mathbb{R}^{n}} \bigl[M_{\alpha,\bar{\varphi }}\hbar (x) \bigr]^{p} \chi _{O_{0}}(x)\,\mathrm{d}x \\ & \quad \leq C \int _{O_{0}} \bigl[M_{\alpha,\bar{\varphi }}\hbar (x) \bigr]^{p}\,\mathrm{d}x. \end{aligned}$$

 □

If the kernel function $K_{\alpha }$ and the coefficient functions $\hbar _{I}$ of differential forms are subject to some conditions, the following more important conclusion will be obtained.

### Theorem 2.4

*Let* *φ* *be a Young function on* $[0,+\infty )$, $\hbar =\sum_{I}\hbar _{I}\,\mathrm{d}x_{I}$ *be a differential form on* $\Theta \subset \mathbb{R}^{n}$, *and let all the ordered* *ℓ*-*tuples* *I* *satisfy* $\hbar _{I}\in L^{\infty }_{c}$. *Suppose that* $F_{\alpha }$ *is a fractional convolution operator on differential forms and its kernel function* $K_{\alpha }$ *satisfies* $K_{\alpha }\in S_{\alpha }\cap H_{\alpha,\varphi }$ *and* $K_{\alpha }\in C^{\infty }_{0}(\Theta )$, *where* $C^{\infty }_{0}(\Theta )$ *stands for all the* $C^{\infty }$ *functions with compactly supported sets in* Θ *and* $0<\alpha <n$. *Then there exists a constant* *C* *such that*

2.13 $$ \bigl\Vert F_{\alpha }\hbar -(F_{\alpha }\hbar )_{O} \bigr\Vert _{p,O}\leq C\operatorname{diam}(O)\vert O \vert \bigl\Vert M_{\alpha,\bar{\varphi }}(\mathrm{d}\hbar ) \bigr\Vert _{p,O} $$

*for any* $1< p<\infty $ *and all the balls with* $O\subset \mathbb{R}^{n}$.

### Proof

By the exterior derivative operator *d* and the fractional convolution operator $F_{\alpha }$, we obtain that

2.14 $$\begin{aligned} &d\hbar =\sum_{I}\sum ^{n}_{k=1}\frac{{\partial \hbar _{I}(x)}}{{\partial x_{k}}}\,\mathrm{d}x_{k}\wedge\mathrm{d}x_{I}, \\ &F_{\alpha }(d\hbar )=\sum_{I}\sum ^{n}_{k=1} \biggl( \int _{\mathbb{R}^{n}}K_{\alpha }(x-y)\frac{{\partial \hbar _{I}(y)}}{{\partial y_{k}}}\,\mathrm{d}y \biggr)\,\mathrm{d}x_{k}\wedge\mathrm{d}x_{I} \end{aligned}$$

and

2.15 $$ dF_{\alpha }(\hbar )=\sum_{I}\sum ^{n}_{k=1}\frac{{\partial h_{I}(x)}}{{\partial x_{k}}}\,\mathrm{d}x_{k} \wedge\mathrm{d}x_{I}, $$

where

2.16 $$ h_{I}(x)= \int _{\mathbb{R}^{n}}K_{\alpha }(x-y)\hbar _{I}(y)\,\mathrm{d}y. $$

According to (1.7)–(1.9), it follows that

2.17 $$ \bigl\Vert F_{\alpha }\hbar -(F_{\alpha }\hbar )_{O} \bigr\Vert _{p,O}= \bigl\Vert T \bigl(d(F_{\alpha }\hbar ) \bigr) \bigr\Vert _{p,O}\leq C_{1}\operatorname{diam}(O)\vert O \vert \bigl\Vert d(F_{\alpha }\hbar ) \bigr\Vert _{p,O}. $$

Now we will give the $L^{p}$-norm estimation of $d(F_{\alpha }\hbar )$. With $K_{\alpha }\in C^{\infty }_{0}(\Theta )$ and considering the definition of the general partial derivative (see [12]), we obtain

2.18 $$\begin{aligned} & \bigl\Vert d(F_{\alpha }\hbar ) \bigr\Vert ^{p}_{p,O} \\ & \quad = \int _{O} \Biggl( \sum_{I}\sum ^{n}_{k=1} \biggl\vert \frac{{\partial h_{I}(x)}}{{\partial x_{k}}} \biggr\vert ^{2} \Biggr) ^{p/2}\,\mathrm{d}x \\ & \quad = \int _{O} \Biggl( \sum_{I}\sum ^{n}_{k=1} \biggl\vert \frac{{\partial \int _{\mathbb{R}^{n}}K_{\alpha }(x-y)\hbar _{I}(y)\,\mathrm{d}y}}{{\partial x_{k}}} \biggr\vert ^{2} \Biggr) ^{p/2}\,\mathrm{d}x \\ & \quad = \int _{O} \Biggl( \sum_{I}\sum ^{n}_{k=1} \biggl\vert \int _{\mathbb{R}^{n}}\frac{{\partial K_{\alpha }(x-y)}}{{\partial x_{k}}}\hbar _{I}(y)\,\mathrm{d}y \biggr\vert ^{2} \Biggr) ^{p/2}\,\mathrm{d}x \\ & \quad = \int _{O} \Biggl( \sum_{I}\sum ^{n}_{k=1} \biggl\vert - \int _{\mathbb{R}^{n}}\frac{{\partial K_{\alpha }(x-y)}}{{\partial y_{k}}}\hbar _{I}(y)\,\mathrm{d}y \biggr\vert ^{2} \Biggr) ^{p/2}\,\mathrm{d}x \\ & \quad = \int _{O} \Biggl( \sum_{I}\sum ^{n}_{k=1} \biggl\vert \int _{\mathbb{R}^{n}}\frac{{\partial \hbar _{I}(y) }}{{\partial y_{k}}}K_{\alpha }(x-y)\,\mathrm{d}y \biggr\vert ^{2} \Biggr) ^{p/2}\,\mathrm{d}x \\ & \quad = \bigl\Vert F_{\alpha }(d\hbar ) \bigr\Vert ^{p}_{p,O}, \end{aligned}$$

that is

2.19 $$ \bigl\Vert d(F_{\alpha }\hbar ) \bigr\Vert _{p,O}= \bigl\Vert F_{\alpha }(d\hbar ) \bigr\Vert _{p,O}. $$

Combining (2.17) and (2.19), we obtain that

2.20 $$\begin{aligned} & \bigl\Vert F_{\alpha }\hbar -(F_{\alpha }\hbar )_{O} \bigr\Vert _{p,O} \\ & \quad \leq C_{1}\operatorname{diam}(O)\vert O \vert \bigl\Vert d(F_{\alpha }\hbar ) \bigr\Vert _{p,O} \\ & \quad =C_{1}\operatorname{diam}(O)\vert O \vert \bigl\Vert F_{\alpha }(d\hbar ) \bigr\Vert _{p,O} \\ & \quad \leq C_{1}\operatorname{diam}(O)\vert O \vert \bigl\Vert M_{\alpha,\bar{\varphi }}(d\hbar ) \bigr\Vert _{p,O}. \end{aligned}$$

 □

Since a new function is obtained when the differential form is taken as a model, we can get a global inequality in the $L^{p}(m)$ domain with Theorem 2.4. Now recall the definition of the $L^{p}(m)$ domain introduced by Staples (see [13]).

### Definition 2.1

Let Θ be a real subdomain in $\mathbb{R}^{n}$. If, for all the functions $f\in L^{p}_{\textrm{loc}}(\Theta )$, there exists a constant *C* such that

2.21 $$ \vert \Theta \vert ^{-1/p}\Vert f-f_{O_{0}} \Vert _{p,\Theta }\leq C\sup_{O\subset \Theta }\vert O \vert ^{-1/p}\Vert f-f_{O} \Vert _{p,O}, $$

then Θ is called an $L^{p}(m)$-average domain, where $O_{0}$ is a fixed ball of Θ and $p\geq 1$.

### Theorem 2.5

*Let* *φ* *be a Young function on* $[0,+\infty )$, $\hbar =\sum_{I}\hbar _{I}\,\mathrm{d}x_{I}$ *be a differential form on* $\Theta \subset \mathbb{R}^{n}$, *and let all the ordered* *ℓ*-*tuples* *I* *satisfy* $\hbar _{I}\in L^{\infty }_{c}$. *Suppose that* $F_{\alpha }$ *is a fractional convolution operator on differential forms and its kernel function* $K_{\alpha }$ *satisfies* $K_{\alpha }\in S_{\alpha }\cap H_{\alpha,\varphi }$ *and* $K_{\alpha }\in C^{\infty }_{0}(\Theta )$, *where* $0<\alpha <n$. *Then there exists a constant* *C* *such that*

2.22 $$ \bigl\Vert F_{\alpha }\hbar -(F_{\alpha }\hbar )_{O_{0}} \bigr\Vert _{p,\Theta }\leq C\vert \Theta \vert \operatorname{diam}(\Theta ) \bigl\Vert M_{\alpha,\bar{\varphi }}d\hbar (x) \bigr\Vert _{p,\Theta } $$

*for any* $1< p<\infty $ *and* $O_{0}$ *is a fixed ball in* Θ.

### Proof

By the definition of the $L^{p}(m)$-average domain and noticing that $1-1/p\geq 0$, we have

2.23 $$\begin{aligned} & \bigl\Vert F_{\alpha }\hbar -(F_{\alpha }\hbar )_{O_{0}} \bigr\Vert _{p,\Theta } \\ & \quad \leq C_{1} \vert \Theta \vert ^{1/p}\sup _{O\subset \Theta }\vert O \vert ^{-1/p} \bigl\Vert F_{\alpha }\hbar -(F_{\alpha }\hbar )_{O} \bigr\Vert _{p,O} \\ & \quad \leq C_{1} \vert \Theta \vert ^{1/p}\sup _{O\subset \Theta }\vert O \vert ^{-1/p}C_{2} \operatorname{diam}(O)\vert O \vert \bigl\Vert M_{\alpha,\bar{\varphi }}(d\hbar ) \bigr\Vert _{p,O} \\ & \quad \leq C_{3} \vert \Theta \vert ^{1/p}\sup _{O\subset \Theta }\vert O \vert ^{1-1/p}\operatorname{diam}(O) \bigl\Vert M_{\alpha,\bar{\varphi }}(d\hbar ) \bigr\Vert _{p,O} \\ & \quad \leq C_{3} \vert \Theta \vert ^{1/p}\sup _{O\subset \Theta }\vert \Theta \vert ^{1-1/p}\operatorname{diam}( \Theta ) \bigl\Vert M_{\alpha,\bar{\varphi }}(d\hbar ) \bigr\Vert _{p,\Theta } \\ & \quad =C_{3} \Theta \vert \operatorname{diam}(\Theta ) \bigl\Vert M_{\alpha,\bar{\varphi }}(d\hbar ) \bigr\Vert _{p,\Theta }. \end{aligned}$$

 □

## The Lipschitz and BMO norm inequalities for the fractional convolution operator

It is well known that Lipschitz and BMO norms are two kinds of important norms in differential forms, which can be found in [14]. Now we recall these definitions as follows. Let $\hbar \in L^{1}_{\textrm{loc}}(\Theta,\bigwedge^{\ell }),\ell =0,1,\ldots,n$. We write $\hbar \in \operatorname{locLip}_{k}(\Theta,\bigwedge^{\ell }),0\leq k \leq 1$, if

3.1 $$ \Vert \hbar \Vert _{\operatorname{locLip}_{k},\Theta }=\sup_{\rho O\subset \Theta }\vert O \vert ^{-(n+k)/n}\Vert \hbar -\hbar _{O} \Vert _{1,O}< \infty $$

for some $\rho \geq 1$.

Further, we write $\operatorname{Lip}_{k}(\Theta,\bigwedge^{\ell })$ for those forms whose coefficients are in the usual Lipschitz space with exponent *k* and write $\Vert \hbar \Vert _{\operatorname{Lip}_{k},\Theta }$ for this norm. Similarly, for $\hbar \in L^{1}_{\textrm{loc}}(\Theta,\bigwedge^{\ell }),\ell =0,1,\ldots,n$, we write $\hbar \in BMO(\Theta,\bigwedge^{\ell })$ if

3.2 $$ \Vert \hbar \Vert _{\star,\Theta }=\sup_{\rho O\subset \Theta }\vert O \vert ^{-1}\Vert \hbar -\hbar _{O} \Vert _{1,O}< \infty $$

for some $\rho \geq 1$.

When *ħ* is a 0-form, Eq. (3.2) reduces to the classical definition of $BMO(\Theta )$.

### Lemma 3.1

(see [10])

*Let* $0< p,q<\infty $ *and* $1/s=1/p+1/q$. *If* *f* *and* *g* *are two measurable functions on* $\mathbb{R}^{n}$, *then*

3.3 $$ \Vert fg \Vert _{s,\Theta }\leq \Vert f \Vert _{p,\Theta }\Vert g \Vert _{q,\Theta } $$

*for any* $\Theta \subset \mathbb{R}^{n}$.

### Theorem 3.1

*Let* *φ* *be a Young function on* $[0,+\infty )$, $\hbar =\sum_{I}\hbar _{I}\,\mathrm{d}x_{I}$ *be a differential form on* $\Theta \subset \mathbb{R}^{n}$, *and let all the ordered* *ℓ*-*tuples* *I* *satisfy* $\hbar _{I}\in L^{\infty }_{c}$. *Suppose that* $F_{\alpha }$ *is a fractional convolution operator on differential forms and its kernel function* $K_{\alpha }$ *satisfies* $K_{\alpha }\in S_{\alpha }\cap H_{\alpha,\varphi }$ *and* $K_{\alpha }\in C^{\infty }_{0}(\Theta )$, *where* $0<\alpha <n$. *Then*, *for any* $1< p<\infty $, *there exists a constant* *C* *such that*

3.4 $$ \Vert F_{\alpha }\hbar \Vert _{\operatorname{locLip}_{k}, \Theta }\leq C \bigl\Vert M_{\alpha,\bar{\varphi }}(d\hbar ) \bigr\Vert _{p, \Theta }, $$

*where* *k* *is a constant with* $0\leq k\leq 1$.

### Proof

By Theorem 2.4, we obtain

3.5 $$ \bigl\Vert F_{\alpha }\hbar -(F_{\alpha }\hbar )_{O} \bigr\Vert _{p,O}\leq C\vert O \vert \operatorname{diam}(O) \bigl\Vert M_{\alpha,\bar{\varphi }}d\hbar (x) \bigr\Vert _{p,O}. $$

By Lemma 3.1 with $1=1/p+(p-1)/p$, for any ball with $O(O\subset \Theta )$, we have

3.6 $$\begin{aligned} & \bigl\Vert F_{\alpha }\hbar -(F_{\alpha }\hbar )_{O} \bigr\Vert _{1, O} \\ & \quad = \int _{O} \bigl\vert F_{\alpha }\hbar -(F_{\alpha } \hbar )_{O} \bigr\vert \,\mathrm{d}x \\ & \quad \leq \biggl( \int _{O} \bigl\vert F_{\alpha }\hbar -(F_{\alpha } \hbar )_{O} \bigr\vert ^{p}\,\mathrm{d}x \biggr) ^{1/p} \biggl( \int _{O}1^{\frac{p}{{p-1}}}\,\mathrm{d}x \biggr) ^{(p-1)/p} \\ & \quad =\vert O \vert ^{(p-1)/p} \bigl\Vert F_{\alpha }\hbar -(F_{\alpha }\hbar )_{O} \bigr\Vert _{p,O} \\ & \quad =\vert O \vert ^{1-1/p} \bigl\Vert F_{\alpha }\hbar -(F_{\alpha }\hbar )_{O} \bigr\Vert _{p,O} \\ & \quad \leq \vert O \vert ^{1-1/p}C_{1}\vert O \vert \operatorname{diam}(O) \bigl\Vert M_{\alpha,\bar{\varphi }}d\hbar (x) \bigr\Vert _{p,O} \\ & \quad \leq C_{2}\vert O \vert ^{2-1/p+1/n} \bigl\Vert M_{\alpha,\bar{\varphi }}d\hbar (x) \bigr\Vert _{p,O}. \end{aligned}$$

By the definition of the Lipschitz norm and $2-1/p+1/n-1-k/n=1-1/p+1/n-k/n>0$, we obtain

3.7 $$\begin{aligned} &\Vert F_{\alpha }\hbar \Vert _{\operatorname{locLip}_{k},\Theta } \\ & \quad =\sup_{\rho O\subset \Theta }\vert O \vert ^{-(n+k)/n} \bigl\Vert F_{\alpha }\hbar -(F_{\alpha }\hbar )_{O} \bigr\Vert _{1,O} \\ & \quad =\sup_{\rho O\subset \Theta }\vert O \vert ^{-1-k/n} \bigl\Vert F_{\alpha }\hbar -(F_{\alpha }\hbar )_{O} \bigr\Vert _{1,O} \\ & \quad \leq \sup_{\rho O\subset \Theta }\vert O \vert ^{-1-k/n}C_{2} \vert O \vert ^{2-1/p+1/n} \bigl\Vert M_{\alpha,\bar{\varphi }}d\hbar (x) \bigr\Vert _{p,O} \\ & \quad =\sup_{\rho O\subset \Theta }C_{2}\vert O \vert ^{1-1/p+1/n-k/n} \bigl\Vert M_{\alpha,\bar{\varphi }}d\hbar (x) \bigr\Vert _{p,O} \\ & \quad \leq \sup_{\rho O\subset \Theta }C_{2}\vert \Theta \vert ^{1-1/p+1/n-k/n} \bigl\Vert M_{\alpha,\bar{\varphi }}d\hbar (x) \bigr\Vert _{p,O} \\ & \quad \leq C_{3}\sup_{\rho O\subset \Theta } \bigl\Vert M_{\alpha,\bar{\varphi }}d\hbar (x) \bigr\Vert _{p,O} \\ & \quad \leq C_{3} \bigl\Vert M_{\alpha,\bar{\varphi }}d\hbar (x) \bigr\Vert _{p,\Theta }. \end{aligned}$$

 □

### Lemma 3.2

(see [14])

*If the differential form* $\hbar \in \operatorname{locLip}_{k}(\Theta, \Lambda ^{\ell })$, $\ell =0,1,\ldots,n$, $0\leq k\leq 1$, *is defined in a bounded convex domain* Θ, *then* $\hbar \in BMO(\Theta, \Lambda ^{\ell })$ *and there exists a constant* *C* *such that*

3.8 $$ \Vert \hbar \Vert _{\star,\Theta }\leq C\Vert \hbar \Vert _{\operatorname{locLip}_{k},\Theta }. $$

By Theorem 3.1 and Lemma 3.2, we get the following conclusion.

### Theorem 3.2

*Let* *φ* *be a Young function on* $[0,+\infty )$, $\hbar =\sum_{I}\hbar _{I}\,\mathrm{d}x_{I}$ *be a differential form on* $\Theta \subset \mathbb{R}^{n}$, *and let all the ordered* *ℓ*-*tuples* *I* *satisfy* $\hbar _{I}\in L^{\infty }_{c}$. *Suppose that* $F_{\alpha }$ *is a fractional convolution operator on differential forms and its kernel function* $K_{\alpha }$ *satisfies* $K_{\alpha }\in S_{\alpha }\cap H_{\alpha,\varphi }$ *and* $K_{\alpha }\in C^{\infty }_{0}(\Theta )$, *where* $0<\alpha <n$. *Then*, *for any* $1< p<\infty $, *there exists a constant* *C* *such that*

3.9 $$ \Vert F_{\alpha }\hbar \Vert _{\star, \Theta }\leq C \bigl\Vert M_{\alpha,\bar{\varphi }}(d\hbar ) \bigr\Vert _{p, \Theta }. $$

## Applications

With regard to the applications of the fractional convolution operator, we will point out that Theorem 2.2 has different expression forms.

### Definition 4.1

(see [7])

Let $K_{\alpha }(x)$ be a function defined on $\mathbb{R}^{n}$, if there exist two constants $c\geq 1$ and $C>0$ such that

4.1 $$ \bigl\vert K_{\alpha }(x-y)-K_{\alpha }(x) \bigr\vert \leq C \frac{\vert y \vert }{\vert x \vert ^{n+1-\alpha }}, \quad \vert x \vert >c\vert y \vert , $$

then the kernel function $K_{\alpha }$ is said to satisfy the $H^{\ast }_{\alpha,\infty }$-condition.

### Lemma 4.1

(see [7])

*Let* *φ* *be any Young function defined on* $[0,+\infty )$, *then* $H^{\ast }_{\alpha,\infty }\subset H_{\alpha, \varphi }$.

### Theorem 4.1

*Let* $K_{\alpha }(x)=\frac{{1}}{{\vert x \vert ^{n-\alpha }}}$ *and* $0\leq \alpha < n$, *then* $K_{\alpha }\in S_{\alpha }\cap H_{\alpha,\varphi }$.

### Proof

Firstly prove that $K_{\alpha }\in S_{\alpha }$. By the definition of $K_{\alpha }$, we have

4.2 $$ \int _{\vert x \vert \sim s} \bigl\vert K_{\alpha }(x) \bigr\vert \mathrm{d}x\leq \int _{O(0,2s)}\frac{{1}}{{\vert x \vert ^{n-\alpha }}}\,\mathrm{d}x\leq \sigma _{n}(2s)^{n}\cdot (s)^{\alpha -n}=2^{n}\sigma _{n}s^{\alpha }, $$

where $\sigma _{n}$ is the volume of a unit sphere *n* in $\mathbb{R}^{n}$. Thus $K_{\alpha }\in S_{\alpha}$.

Secondly prove that $K_{\alpha }\in H_{\alpha,\varphi }$. According to Lemma 4.1, we only need to prove that $K_{\alpha }\in H^{\ast }_{\alpha,\infty }$. If we choose $\vert x \vert >2\vert y \vert (c=2\geq 1)$ and $y\neq O=(0,\ldots,0)$ (for $y=O$, it is clearly established), it follows that

$$ \vert x-y \vert /\vert x \vert \in \textstyle\begin{cases} (\frac{1}{2},1), &x, y \text{ each component has the same sign;} \\ (1,\frac{3}{2}), &x, y \text{ each component has the different sign,} \end{cases} $$

where $x=(x_{1},\ldots,x_{n}),y=(y_{1},\ldots,y_{n})$. Considering that each component of *x*, *y* is greater than zero, other cases may be considered similarly. By Lagrange’s mean value theorem

4.3 $$\begin{aligned} & \bigl\vert K_{\alpha }(x-y)-K_{\alpha }(x) \bigr\vert \\ & \quad = \bigl\vert \vert x-y \vert ^{\alpha -n}- \vert x \vert ^{\alpha -n} \bigr\vert \\ & \quad =\vert x \vert ^{\alpha -n} \biggl\vert \biggl( \frac{\vert x-y \vert }{\vert x \vert } \biggr)^{\alpha -n}-1 \biggr\vert \\ &\quad \leq (n-\alpha )\vert x \vert ^{\alpha -n} \biggl( \frac{\vert y \vert }{\vert x \vert } \biggr) \bigl(\vert \xi \vert \bigr)^{\alpha -n-1} \\ &\quad \leq 2^{n+1-\alpha }(n-\alpha )\frac{\vert y \vert }{\vert x \vert ^{n+1-\alpha }}, \end{aligned}$$

where

$$\begin{aligned}& \vert x \vert =\sqrt{\sum^{n}_{i=1}x^{2}_{i}}, \xi =(\xi _{1},\ldots,\xi _{n}), \\& \frac{1}{2}< \min \biggl\{ \frac{\vert x-y \vert }{\vert x \vert },1 \biggr\} < \vert \xi \vert < \max \biggl\{ \frac{\vert x-y \vert }{\vert x \vert },1 \biggr\} < \frac{3}{2}. \end{aligned}$$

Thus $K_{\alpha }\in H^{\ast }_{\alpha,\infty }$. □

Theorems 2.2 and 4.1 yield the following.

### Theorem 4.2

*Let* *φ* *be any Young function defined on* $[0,+\infty )$ *and* $K_{\alpha }(x)=\frac{{1}}{{\vert x \vert ^{n-\alpha }}}$, *then the fractional convolution operator* $F_{\alpha }$ *in* (1.1) *becomes the classical Riesz potential operator*

4.4 $$ I_{\alpha }\hbar (x)=\sum_{I} \biggl( \int _{\mathbb{R}^{n}}\frac{1}{{\vert x-y \vert ^{n-\alpha }}}\hbar _{I}(y)\,\mathrm{d}y \biggr)\,\mathrm{d}x_{I}, $$

*where* $\hbar =\sum_{I}\hbar _{I}\,\mathrm{d}x_{I}$ *is a differential form in* $\mathbb{R}^{n}$ *and such that* $\hbar _{I}\in L^{\infty }_{c}$ *for all the ordered* *ℓ*-*tuples I*. *Then there exists a constant* *C* *such that*

4.5 $$ \int _{\mathbb{R}^{n}} \bigl\vert I_{\alpha }\hbar (x) \bigr\vert ^{p}w(x)\,\mathrm{d}x\leq C \int _{\mathbb{R}^{n}} \bigl[M_{\alpha,\bar{\varphi }}\hbar (x) \bigr]^{p}w(x)\,\mathrm{d}x $$

*for any* $0< p<\infty $ *and* $w\in A_{\infty }$.

### Lemma 4.2

(see [7])

*Denote by* $S^{n-1}$ *the unit sphere of* $\mathbb{R}^{n}$, Ω *is a homogeneous function defined on* $S^{n-1}$ *with* $\Omega (x)=\Omega (x')$ *and the kernel function* $K_{\alpha }(x)=\Omega (x)/\vert x \vert ^{n-\alpha }(x\neq 0)$, *where* $x'=x/\vert x \vert (x\neq 0)$. *Given a Young function* *φ*, *we define the* $\L^{\varphi }$-*modulus of continuity of* Ω *as*

4.6 $$ \varpi _{\varphi }(t)=\sup_{\vert y \vert \leq t}\nparallel \Omega (\cdot+y)-\Omega (\cdot )\bigr\Vert _{\varphi (S^{n-1})} $$

*and write* $\Omega \in \L^{\varphi }(S^{n-1})$. *If*

4.7 $$ \int ^{1}_{0}\varpi _{\varphi }(t) \frac{{\mathrm{d}t}}{t}< \infty, $$

*then* $K_{\alpha }\in S_{\alpha }\cap H_{\alpha,\varphi }$.

By Theorem 2.2 and Lemma 4.2, we have the following.

### Theorem 4.3

*Let* *φ* *be a Young function*, Ω *be a homogeneous function in* $S^{n-1}$ *with* $\Omega (x)=\Omega (x')$ *and* $\Omega \in \L^{\varphi }(S^{n-1})$. *Suppose that* $F_{\alpha }$ *is the fractional convolution operator with its kernel function* $K_{\alpha }(x)=\Omega (x)/\vert x \vert ^{n-\alpha }$. *Let* $\hbar =\sum_{I}\hbar _{I}\,\mathrm{d}x_{I}$ *be a differential form in* $\mathbb{R}^{n}$ *with* $\hbar _{I}\in L^{\infty }_{c}$ *for all the ordered* *ℓ*-*tuples I*. *If* $\int ^{1}_{0}\varpi _{\varphi }(t)\frac{{\mathrm{d}t}}{t}<\infty $, *then there exists a constant* *C* *such that*

4.8 $$ \int _{\mathbb{R}^{n}} \bigl\vert F_{\alpha }\hbar (x) \bigr\vert ^{p}w(x)\,\mathrm{d}x\leq C \int _{\mathbb{R}^{n}} \bigl[M_{\alpha,\bar{\varphi }}\hbar (x) \bigr]^{p}w(x)\,\mathrm{d}x $$

*for any* $0< p<\infty $ *and* $w\in A_{\infty }$.
